# Language after Hemispherotomy in Rasmussen Syndrome

**DOI:** 10.15844/pedneurbriefs-30-2-4

**Published:** 2016-02

**Authors:** Sophia Varadkar, Martin Tisdall

**Affiliations:** 1 Neurosciences Unit, Great Ormond Street Hospital for Children NHS Foundation Trust and UCL Institute of Child Health, London, UK

**Keywords:** Rasmussen Syndrome, Hemispherotomy, Hemispherectomy, Language

## Abstract

Investigators from Rothschild Foundation Hospital in Paris report language outcome in children after (left) dominant-hemisphere hemispherotomy for Rasmussen Syndrome.

Investigators from Rothschild Foundation Hospital in Paris report language outcome in children after (left) dominant-hemisphere hemispherotomy for Rasmussen Syndrome. Six children underwent surgery at ages 4.1 to 8.4 years. Preceding duration of epilepsy was short, mean of 1.2 years. Four experienced pre-operative aphasia with decreased verbal comprehension indices (VCI) (4/6) and performance reasoning index (5/6). Mean duration of follow-up was 5.6 years. Longitudinal follow-up of cognition and speech is reported. Post-operatively VCI normalized over 3-8.3 years. The authors describe a specific linguistic profile: normal verbal comprehension, and weakness of grammatical judgment, word repetition, statement production, semantic verbal fluency and metaphonological abilities. Working memory scores were also weak, though not meeting statistical significance.

The authors suggest this study emphasizes the ability of the right hemisphere to functionally reorganize language over a long period of time following surgery, support decision making toward hemispherotomy in left-sided Rasmussen syndrome and can aid post-operative follow-up and rehabilitation strategies. [1]

COMMENTARY. Rasmussen syndrome is a rare chronic progressive neurological disease of presumed immune basis characterized by unilateral inflammation and atrophy of a cerebral hemisphere, drug-resistant focal epilepsy, progressive hemiplegia and cognitive decline [2]. To date medical therapies are imperfect and can but alleviate symptoms. Only surgery, specifically hemispherotomy (disconnection of the affected hemisphere) halts the disease. Surgery gives a high chance of seizure freedom (70-80%) and cognitive stabilization, but at the cost of inflicting hemiplegia, homonymous hemianopia and in language-dominant hemisphere disease, aphasia. These deficits may be present preoperatively, with variable severity and alacrity. Children adapt to hemianopia, walk again though with an ankle splint, but do not recover upper limb fine-motor function. Though it is known that language function can be sub-served by one non-dominant hemisphere alone, decisions around timing of surgery with regard to language recovery are more difficult.

Careful pre-operative neuropsychological testing and in the cooperative child language functional MRI can lateralise language, making Wada testing, long deemed the gold-standard, no longer essential. Interval imaging may show transfer/re-localisation of language dominance to the contra-lateral hemisphere over the disease course, giving the epilepsy surgery team more confidence in the prospects of post-hemispherotomy language recovery and more confidence to proceed. Earlier onset seizures may increase the chances of this transfer but it is not known if earlier surgery could better drive this at a younger age with more retained neural plasticity, or whether it is better to allow function to become established naturally in the unaffected hemisphere, accepting that cognitive decline proceeds inexorably [3].

The present study is encouraging; all children regained speech and long-term follow-up demonstrated on-going language recovery. Rehabilitation is essential after surgery and the patterns of language recovery delineated here can also inform therapy programs and goal-setting. Further, the parallel to the cognitive gains 5-years out from temporal lobe surgery, remind us the importance of long-term follow-up studies in informing our decision-making in the acute disease setting [4].
